# Practical applicability of genetics for the prevention and treatment of hypertension

**DOI:** 10.1111/jch.14400

**Published:** 2021-12-28

**Authors:** Alexandre Sérgio Silva

**Affiliations:** ^1^ Federal University of Paraíba/Department of Physical Education Cidade Universitária João Pessoa‐PB Brazil

## Abstract

Zou and colleagues are publishing in this issue of The Journal of Clinical Hypertension, result of one of their studies in which they found that human corin genetic polymorphisms is involved in blood pressure control, more specifically in salt sensitivity. It is being published in this journal shortly after a literature review indicated another 18 genes were also involved in salt sensitivity, however corin gene. This dynamism of newly discovered genes shows the complexity of studying the genetic control of arterial hypertension and explains its known preliotropic characteristic. In this commentary, the study by Zou and colleagues is placed in the context of recent evidence on the genetics of high blood pressure and the future perspectives resulting from this and other studies are presented in the context of the clinical application of genetics in the prevention and treatment of high blood pressure.

In this issue of The Journal of Clinical Hypertension, an article is being published that reports the results of a study conducted by Zou and 28 collaborators^1^ from several research institutions, in which they found a new gene associated with blood pressure control, particularly in sensitivity to sodium intake. The multicentric effort of this work both recalls and follows the joint effort perspective that was made in the human genome mapping project,^2^ one of the most impactful scientific activities of the late 20th century and early 21st century.

These two works with multicentric characteristics are examples of the need for joint efforts to deal with the complexity of studying the interactions of more than 25 000 human genes. An example of the complexity of the genetic interaction to control blood pressure is well shown just in article published by Zou,^1^ which demonstrated that polymorphism in the corin gene was associated with salt sensitivity and development of hypertension. This study is being published only two years after a literature review pointed out 21 genes involved in hormonal control of the blood pressure since the genome project until then, of which 18 of them were associated with salt‐sensitivity hypertension.^3^ Among these 18 genes discovered, the genetic polymorphism of corin had not been mentioned. This state of the art easily convinces that the search for genes involved in the blood pressure response to salt intake cannot yet be considered to be completeness.

Controlling blood pressure is even more complex when considering the plead of other biological mechanisms involved in blood pressure control, such as autonomic nervous activity,^4^ endothelial function,^5^ oxidative stress, and systemic inflammation.^6^ It should be taken into account that environmental and even socioeconomic variables interact to determine an adequate blood pressure control or the development of a hypertensive state. Thus, the challenge of finding answers to the genetic‐environment relationship is even greater.

Despite a good and growing availability of antihypertensive drugs, as well as non‐drug therapies such as physical training^7^ and some foods,^8^, ^9^ the success rates in antihypertensive treatment still remains below 50%.^10^ So, it is precisely in this scenario that the understanding of the genetic aspects involved in blood pressure control may represent a possibility of improvement in this results. For this, a plausible possibility is precisely to increase our knowledge on responsiveness to treatments modulated by genetic determinants. In the case of pharmacological treatment, a relationship of genetic ‐ polymorphism dependence have been shown. In the review by Rysz and colleagues,^11^ are presented studies that showed that genetic polymorphisms are associated with treatment responsiveness for each of the antihypertensive drug classes (calcium channel blockers, nine studies; angiotensin II receptor blockers, nine studies; beta‐blockers, nine studies; beta blockers, nine studies; adrenergic blockers, 10 studies; and diuretics, nine studies).

The discovery of genetic ‐ polymorphism dependence of responses to antihypertensive treatments, however, still does not seem enough. Because of the multifactorial nature of arterial hypertension, we must understand that the genome project represented only a starting point that provided us researchers with a basic tool to unravel how we can translate genetic knowledge into the treatment of arterial hypertension. While works such as those by Zou and colleagues ^1^ have added a new gene involved in blood pressure responses to salt intake, it is possible that other studies will discover other genes involved in this same phenomenon and many other genes involved in the multiple other mechanisms involved in the control of blood pressure. Given this complexity, it seems that the most plausible path for the future of this line of investigation is the advance from the single nucleotide polymorphism level to the omics level.

While genomics is potentially promising in terms of contributing to the advancement of gene‐gene interaction, at the same time it suggests the need to advance to other areas of cell and molecular biology, such as epigenomics, transcriptomics, proteomics, and metabolomics. Epigenomics has the potential to clarify how environmental factors modify innate genetic tendencies to control blood pressure; transcriptomics, proteomics and metabolomics have the potential to indicate drug treatments that attenuate cellular responses promoted by genomic interactions associated with the pathophysiology of arterial hypertension, as drugs can act by stimulating or inhibiting transcription processes, micro RNA activity and protein formation involved in blood pressure control. Applications of pharmacogenomics can already be considered a plausible possibility to increase the antihypertensive success rate from clinical drug intervention, as argued in the review by Arnett and Claas.^12^ Additionally, we already have evidence that both physical training^13^ and certain nutrients^14^ interfere in the control of gene expressions that contribute to the increase in blood pressure.

Advancing to the level of omics does not exclude the need for studies with single nucleotide polymorphism studies like the one by Zou and colleagues. ^1^ These studies effectively contribute to the advancement of knowledge through the identification of genes involved in blood pressure control. Data from this study, from other genetic polymorphisms that have already been discovered and others that have yet to be unveiled, will serve to form an interaction network that may explain the genetic influence on the development and treatment of hypertension. The relevance of this theme has been noted by several research groups that have devoted attention to the study of single nucleotide polymorphism that may be affecting the etiology of arterial hypertension^15^ responses to sodium intake,^16^, ^17^ only among more recent articles published in the Journal of Clinical Hypertension.

Finally, while single nucleotide polymorphism studies offer the links (set of genes) that must be linked to understand the complex chain of genetic interaction involved in controlling hypertension, epigenomics can shed light on how the environment interferes with innate genetic programming to better explain this environment‐gene interaction; the proteomics, transcriptomics, and metabolomics can shed light on how we can interfere with cellular mechanisms through nutritional, pharmacological or lifestyle aspects so that we have a more complete mastery of this complex system of blood pressure control.

## CONFLICT OF INTEREST

The author has no competing interests.
